# The British Medical Association Meeting

**Published:** 1910-08-06

**Authors:** 


### THE BRITISH MEDICAL ASSOCIATION MEETING.

### SOME NOTES AND IMPRESSIONS.

By a Correspondent.

The *locale* of the seventy-eighth annual meeting of the B.M.A. was certainly a good one. True, the whole thing was not under one roof, the President giving his address in a hall in Great Portland Street, while the sections for which there was no room in the Imperial Institute itself found accommodation at the Imperial College of Science and the Old College of Science respectively. In the last two cases, all that was necessitated was a walk across the road. Moreover, the numbers becoming distributed, all crowding was avoided. Everywhere conspicuous, plainly worded notices (what, however—off-hand—is a "lady invigilator"?) made one's way as easy to find as in a tube station. Nor must there be forgotten the abundant literature to be procured gratis, literature which ranged in subject from 'bus-fares to medical history—illustrating, by the way, the analogy that must exist between the intellect of the modern journalist and the Nasmyth steam hammer—and which showed an ability and a careful preparation too often absent from the many discourses heard.

#### President's Address.

Mr. Butlin's address, which for most members marked the beginning of the Congress, was a rather notable effort of memory; for though he had necessarily to give many dates, names, and figures, he used no notes at all. Beginning by recalling the many important scientific announcements made at previous annual meetings, he then traced in detail the labours of the Association in the cause of medical reform, frequent mention being, of course, made in this connection of the name of Sir Charles Hastings. An account followed of some other activities of the Association, as in the encouragement of scientific research and of benevolent provision for needy members and their dependents. As Treasurer, Mr. Butlin remarked, it had been his lot formerly to defend scientific objects from the attacks of utilitarians, and the result justified him, for many such as Victor Horsley, Ferrier, and Washbourn could be mentioned as having won their spurs through the aid of the Association. Mr. Butlin seems fond of Scriptural quotations. The audience, most of whom, especially the Council and representative members on the platform, were in academic dress, gave the President a very sympathetic hearing.

#### Address in Medicine.

The acoustic qualities of the University of London lecture theatre, where Dr. Mitchell Bruce delivered the address in medicine, are not nearly so good as those of St. James's Hall. Moreover, the hour—12.30 p.m.—was not very suitable. Everybody had been hearing speeches the whole morning, and was beginning to think about lunch. A sitting of the physiology section had just taken place, and although Mr. Leonard Hill had been speaking enthusiastically of the benefits of inhalation of oxygen, yet there was not much of that substance left in the atmosphere. However, Dr. Mitchell Bruce, who at a distance resembles curiously Sir Felix Semon, was well worth listening to. He took as his subject the changes in medicine during the last fifteen years, the time elapsed since last the Association met in London. The accretions to our knowledge of etiology would, he thought, mark the period historically. Spite, however, of the opportunities these had made for the laboratory worker, there was yet a sphere of activity—knowledge and control of a patient's idiosyncrasies and surroundings—left untouched to the general practitioner, who, moreover, had, in connection with the facts of morbid inheritance, one unique position of authority. In balanced discrimination, in sagacity, in shrewdness, in competent expression, and in its utter foreignness to the quality of intellectual *élan*, Dr. Bruce's speech was the characteristic performance of an eminent clinician. The vote of thanks was moved from the body of the audience, a thing which may have been an accident, but which has an excellent effect.

#### The Sections.

As regards the various sections, with such a large programme no attempt can be made at anything like a comprehensive survey. Would it not be well to have fewer papers in future—and longer ones? In fifteen minutes surely nothing *eingehend* can be submitted, except in an unattractive, as it were tabloid, form! The quickest speaker could not manage much more than two thousand words in the time, and as few medical men are masters of the art of compression, the result in many instances is superficiality or incompleteness or triviality. Such could not be said, however, of the paper read in the radiology section for Dr. Dominici of Paris, dealing with the results of treatment of carcinoma and sarcoma with radium. Some patients had been sent over from France, and their condition in each case was certainly a vast improvement upon the former state depicted in photograph, epidiascope, and large diagrams of microscopical sections. The need of careful watching for the first signs of relapse was insisted upon. A good discussion took place in the laryngological section on the bronchoscope, the sovereign utility of which in impaction of foreign bodies within the thorax has been so much testified to lately. The new section of medical sociology was successfully inaugurated. Better qualified writers than the present one will probably deal with the subject of Wednesday morning—that of the economic basis of hospital management. On Friday many spoke on the social aspects of the falling birth-rate, including several ladies. One or two of these dissented from some rhetoric of Dr. Fremantle's. Women, said one, are alternately praised for their energy in entering callings like the medical profession, and admonished to stick to home duties. They would, on the whole, be obliged to their critics if the latter would settle the matter among themselves first. A British audience always warms to the voice of disillusioning common-sense, especially when it comes from a woman, and the meeting signified its assent to this lady's propositions in the usual manner.

The Social Side.

The exhibition of drugs, etc., occupied most of the first floor, a separate room downstairs being given to electrical apparatus. Most of the medical publishers had stalls. There were few provincial firms exhibiting, although Messrs. Reynolds and Branson, of Leeds, were in evidence with outfits for the local application of carbon dioxide snow. The Hygienic Co., Ltd., and Aerators, Ltd., displayed wax figures in aerated baths, the appearance being like bathing in recently drawn soda-water.

Nothing truer can be said of the programme of entertainments than the hackneyed phrase that it was on a lavish scale. Irish hospitality is, of course, hard to equal, but the Ulster members would all admit that the Metropolis acquitted itself most praiseworthily. We practitioners up from the country are under great obligation to the hardworking officials of this meeting. All tastes were catered for, and there must have been nearly as many kinds of diversion available as members attending the meeting. On Thursday afternoon, for instance, a professional billiard match (most kindly provided by Messrs. Thurston of Leicester Square) was watched by half-a-dozen spectators. There was, too, besides the set excursions, which naturally attracted principally domestic parties, a liberal supply of cards giving honorary admission to first-class golf links, courses kept up at great expense and with a long waiting list of applicants for membership. Dr. Haslip won the Ulster cup with a good score.

Lastly, there was the crowd of members, the renewal of friendly acquaintance, the silent—let us hope—noting of the effect of the hand of time upon personal appearance and powers. It was not unpleasant to see the decay, however, of formalism in dress in our profession. This was no black-coated, white-chokered, spectacled, side-whiskered, pretentious looking solemn assembly, but an interested concourse, twice as strenuous, vastly better informed, more careful to be rather than to seem, of twentieth-century men and women intent upon the pursuit of learning and upon enjoying themselves. The latter object may have interfered a little with the former one: in most parts of the Continent we should have had to meet earlier and break up far later. But no one could attend this meeting without having the great fact of rapid progress brought home to him; without having his optimism stirred and his pride in belonging to a profession which, one may say, is rapidly putting the other ones—as now understood—into their proper relative positions to it.

